# The Prevalence of Brucellosis in Different Provinces of Iran during 2013–2015

**Published:** 2019-01

**Authors:** Siavaah CHALABIANI, Mina KHODADAD NAZARI, Nada RAZAVI DAVOODI, Mahdi SHABANI, Masoud MARDANI, Abdolfatah SARAFNEJAD, Ali AKBAR AMIRZARGAR

**Affiliations:** 1. Department of Molecular Diagnostics, Noor Pathobiology Laboratory, Tehran, Iran; 2. Department of Immunology, School of Medicine, Shahid Beheshti University of Medical Sciences, Tehran, Iran; 3. Tropical Infectious Diseases Research Center, Shahid Behshti University of Medical Sciences, Tehran, Iran; 4. Department of Immunology, School of Public Health, Tehran University of Medical Sciences, Tehran, Iran; 5. Department of Immunology, School of Medicine, Tehran University of Medical Sciences, Tehran, Iran; 6. Molecular Immunology Research Center, Tehran University of Medical Sciences, Tehran, Iran

**Keywords:** Brucellosis, Prevalence, Zoonotic, Iran

## Abstract

**Background:**

Brucellosis as a zoonotic disease is widespread among human and animal that it continues to be a major public health problem. Due to the shortage of recent epidemiologic data regarding the brucellosis distribution in Iran, we convinced to evaluate the prevalence of brucellosis in provinces of Iran.

**Methods:**

In this descriptive study, data were collected from brucellosis suspected patients referred to Noor Pathobiology Laboratory, Tehran, Iran from 18 out of 31 provinces of Iran during 2013–2015.

**Results:**

Overall, 2635 out of 17103 attending cases (15.4%) were recognized as brucellosis patients. The most prevalent rate was found in patients aged 20–39 yr old (41%) which of them 67% were male. Patients with brucellosis were significantly diagnosed in spring season (Apr to late May). Among included provinces, Hamadan Province had the highest (25%) prevalence followed by Markazi and Mazandaran with 24.7% and 22.5%, respectively.

**Conclusion:**

Brucellosis is still considered as an important infectious disease with a high prevalence in many provinces of Iran. It is necessary to implement a national brucellosis control program by increasing medical education, public knowledge and various controlling plans for preventing, controlling and eradicating of brucellosis.

## Introduction

Brucellosis is a zoonotic disease transmitted to domestic and wild animals as well as humans. It is reported globally especially in Mexico, the Indian subcontinent, the Mediterranean basin, the Arabian Peninsula and Central and South America (1). Brucellosis has been recognized as a public health issue and one of the major causes of morbidity. A high prevalence in certain geographic areas is well recognized, although largely underestimated (1). The transmission and prevalence of *Brucella* in an area are influenced by some factors, for instance, hygiene, dairy production procedures, husbandry practices, and socioeconomic status (2).

The laboratory diagnosis of brucellosis is essentially performed by detection of anti-*Brucella* anti-bodies and nucleic acid in serum. Different serological assays are applicable now, but for confirming of active infection, at least two serological assays should be considered. At the first step, standard tube agglutination (STA) test (Wright) is done and the obtained results are confirmed by 2-Mercaptoetanole (2ME) test. STA can estimate the total quantity of IgM and IgG, but 2ME test determines the quantity of specific IgG in the acute form of disease (3,4). In Iran, a STA test titer > 1:160 with a 2ME test titer ≥ 1:80, coupled with clinical symptoms, are the most important protocol for brucellosis diagnosis (5,6). Furthermore, ELISA has high sensitivity and specificity in the diagnosis of blood IgM, IgG and IgA specific to *Brucella* (4,7). Besides serological assays, PCR test is also applied for the molecular detection of the *Brucella* microorganism. The *Brucella* DNA is recognizable in serum, blood, pus and tissue samples of infected patients. Currently, Real-time PCR method has been expanded with faster and easier applications in the clinic and a lower contamination probability for detection of *Brucella* (3, 8).

Several brucellosis epidemiology studies have been recently carried out in Iran and other countries (2). A seroprevalence study in the northeast of Iran showed that the prevalence rate of brucellosis in human was 37/100000 during 2002–2006 (9). Brucellosis had a downward trend in 2006 compared to its prevalence in Kurdistan Province of Iran during 2004–2005 (10). Another epidemiologic research in Zanjan Province of Iran indicated that, although increasing of brucellosis prevalence was observed during 2001–2006, this trend has been decreasing from 2006 to 2008 (2). Compatible with this data, the rate of outbreak constituted 42/100000 and 36/100000 in 2006 and 2007, respectively (2). Brucellosis remains endemic in the most areas of the world although it has been eradicated or virtually eradicated from livestock in the northern Europe, Australia, the USA and Canada following lengthy and expensive control programs (11). Brucellosis has the highest incidence in countries such as Palestine (21.5 per 100000), Saudi Arabia (32.8 per 100000), Syria (21.0 per 100000), Iran (29.8 per 100000), Jordan (20.4 per 100000), and Oman (16.6 per 100000). Bahrain and Cyprus have been reported zero incidence (12). In addition, the rate of outbreak decreased in Saudi Arabia to 9 patients per 100000 people in 2006 (12). Economic and social importance of brucellosis makes it necessary to be controlled and prevented.

Due to the shortage of recent epidemiologic data regarding to the brucellosis distribution in Iran, we convinced to evaluate the prevalence of brucellosis in provinces of Iran.

## Materials and Methods

### Patient data gathering

This study was a descriptive cross-sectional survey to find out the prevalence of brucellosis in provinces of Iran. Data were collected from brucellosis suspected patients referred to Noor Pathobiology Laboratory (Tehran, Iran) from 18 out of 31 provinces of Iran from Mar 2013 to Sep 2015. Serologic and molecular assays were performed on each sample based on physicians order.

### Standard tube agglutination (STA) test

The patient’s serum was serially diluted with normal saline in 1/20, 1/40, 1/80, and 1/160 (the denominator was doubled and continued based on positive rate of Rose Bengal test) ratios in multiple test tubes. Then, Wright specific tubular antigen (Pasteur Institute of Iran) was added to each tube and incubated at 37 °C for 24 h. Afterward, the tubes were evaluated for agglutination. Titer 80 or higher is accounted positive for brucellosis. Then, 2ME test was performed for Wright positive samples in order to recognize *Brucella* specific antibody class. For 2ME agglutination test, serum was serially diluted with normal saline and 2ME specific antigen (Pasture Institute of Iran) was added and incubated at 37 °C for 24 h. Last tube with agglutination was considered for 2ME titer report.

### Brucella specific antibody detection by ELISA

Serum obtained from all patients was tested for *Brucella*-specific antibodies using commercial ELISA kit according to manufacturer’s instructions (Pishtazteb Zaman, Iran). Briefly, all sera were appropriately diluted with phosphate-buffered saline (PBS) and 100 μl of each sample was added to antigen-coated 96-well plate and incubated for 30 min at 37 °C. After 5 times washing with wash buffer, 100 μl of HRP-conjugated enzyme was added to the wells and incubated at 37 °C for 30 min. After washing, 100 μl of chromogenic substrate was added to the wells and incubated for 15 min in the dark and room temperature. Finally, 100 μl of stop solution was added to stop reaction and generated color was read at 450 nm by ELISA reader (Hiperion MPR 4+, Germany).

### Brucella recognition by Real-time PCR

Molecular detection of *Brucella* DNA was performed on patients’ serum by Real-time PCR technique. Bacterial DNA was extracted using High Pure PCR Template preparation kit as manufacturer’s instruction (Roche, Germany). Briefly, 200 μl of serum was mixed with equal volume of binding buffer and 40 μl proteinase K and incubated for 10 min at 70 °C. Then, 100 μl isopropanol was added and the mixture was applied to a highly pure filter tube and centrifuge for 1 min at 8000 × g. The filter was washed with 500 μl inhibitor removal buffer and consequently by wash buffer using centrifugation. Flow through was discarded and DNA was eluted by 200 μl elution buffer using centrifugation for 1 min at 8000 × g.

Real-time PCR reactions were performed in a total volume of 25 μl (7 μl of PCR-mix-2-FL and 10 μl of extracted DNA) using commercial kit (AmpliSense *Brucella* spp.-FRT, Moscow, Russia). Hot start DNA polymerase was activated by holding at 95 °C for 5 min and sequentially cycled for 10 times at 95 °C for 10 sec, 65 °C for 25 sec and 72 °C for 10 sec in cycling 1 and cycled for 35 times at 95 °C for 10 sec, 56 °C for 25 sec and 72 °C for 10 sec in cycling 2. Signals were detected at corresponding detectors as FAM/green and JOE/yellow for internal control and *Brucella* specific amplicon, respectively.

### Statistical Analysis

Clinical information about the patients was analyzed by Microsoft Excel (Ver. 2010). Statistical analysis was performed by Prism software (GraphPad Software, Inc, CA, USA).

## Results

The rate of brucellosis prevalence was calculated for each province (Table 1).

**Table 1:** The frequency of brucellosis in studied samples from different provinces of Iran

** Province **	** Frequency of samples **	** Frequency of positive cases **	** Percentage of positive samples **
Hamadan	400	100	25
Markazi	296	73	24.66
Mazandaran	524	118	22.5
Alborz	387	82	21.18
Tehran	8807	1616	18.3
Khuzestan	1511	167	11.05
Lorestan	2890	302	10.44
Esfahan	1834	175	9.5
Zanjan	132	1	0.75
Qazvin	156	1	0.64
Hormozgan	60	0	0
Qom	26	0	0
East-Azerbaijan	32	0	0
West-Azerbaijan	22	0	0
Bushehr	8	0	0
Yazd	8	0	0
Kerman	8	0	0
Gilan	2	0	0
Total	17103	2635	-

Overall, 17103 patients were included aged between 1–90 yr old (mean ± SD=36.85 ± 18.74 yr). Among them, 14279 (83.5%) patients were categorized in adult age group (≥ 20 yr) and 2824 (16.5%) patients were children (< 20 yr). The patients were more subdivided into 4 different subgroups (0–19, 20–39, 40–59 and >60) (Table 2). Gender analysis of patients showed that 10029 (59%) were males (mean age ± SD = 26.78 ± 17.86 yr), and 7074 (41%) were females (mean age=29.48±17.42 yr); the male to female ratio was 2:1.

**Table 2:** The prevalence rate of human brucellosis based on age and gender subdivisions

** Gender **	** Age (yr) **				** Specimen **			** Total **
	0–19	20–39	40–59	> 60	Serum	CSF	Tissue	
Male	500	718	325	158	1478	23	2	1503
Female	241	360	220	113	1118	14	-	1132
Total	741	1078	545	271	2596	37	2	2635

**Fig. 1:**
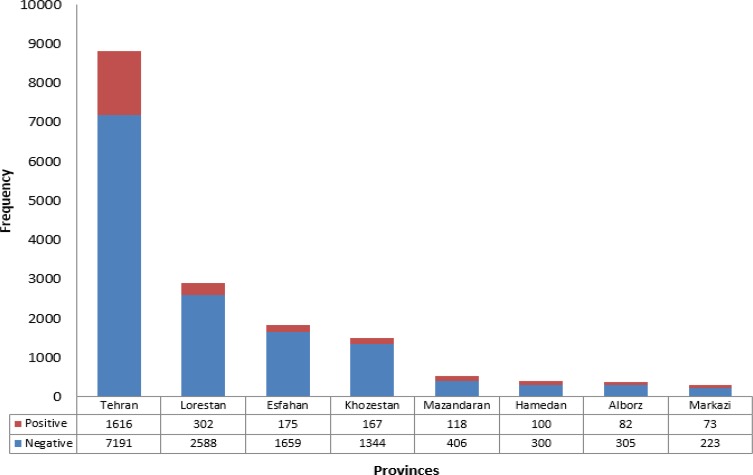
Representative data regarding the prevalence of brucellosis in patients attended to Noor Pathobiology Laboratory during 2013–2015 based on provinces subdivisions

The diagnosis of brucellosis was established by STA, ELISA and PCR tests in 2635 out of 17103(15.4%) of suspected patients (Table 3). Totally, 828/6402 (13%), 1802/10394 (7%) and 5/307 (2%) of evaluated samples were positive using STA, ELISA, and PCR tests, respectively (Table 3). The most prevalent rate of brucellosis was found in patients aged 20–39 yr old (41%) which of them 67% were male. Brucellosis significantly affected working-age male adults compared to females in different age groups (*P*<0.001) (Table 3). Interestingly, the brucellosis frequencies were also different in the studied provinces. In this regard, Hamadan Province had the highest (25%) prevalence followed by Markazi and Mazandaran with 24.7% and 22.5%, respectively during 2013–2015 (Fig. 1).

**Table 3:** Results of STA, ELISA and PCR performed on suspected patients

** STA N(%) **		** ELISA N(%) **		** PCR N(%) **		** Total N(%) **							
Negative	Positive	Negative	Positive	Negative	Positive	Negative	Positive
5574 (87)	828 (13)	8592 (83)	1802 (17)	302 (98)	5 (2)	14468 (85)	2635 (15)
6402		10394		307		17103							

**Fig. 2:**
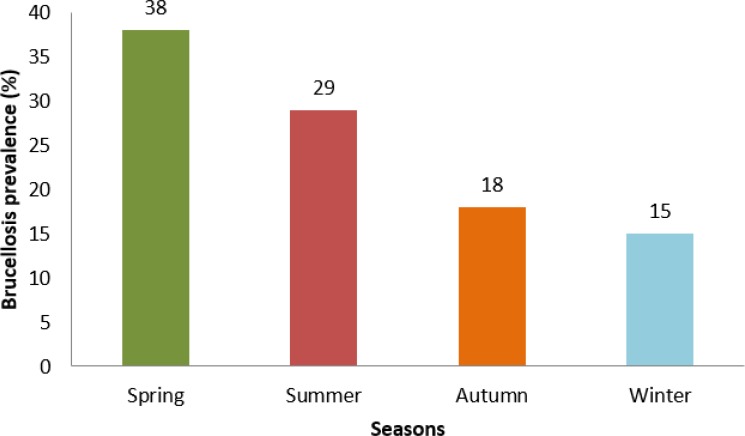
The prevalence of brucellosis in studied patients during 2013–2015 based on seasonal subdivision

All studied cases from Hormozgan, Qom, East-Azerbaijan, West-Azerbaijan, Bushehr, Yazd, Kerman and Gilan were negative for *Brucella* (Table 1). Regarding the seasonal prevalence, the number of infected cases was higher in the spring and summer compared to the autumn and winter (Fig. 2). The peak of disease detection was placed at Apr to late May.

## Discussion

Brucellosis has been considered among the most economically essential zoonotic disease worldwide that its impacts on health and economy completely differ among high-, middle- and low-incoming countries (13). Middle Eastern countries including Iraq, Iran, Syria and Saudi Arabia were reported with the highest annual incidence in 2006 (14). Iran is endemic for brucellosis where its average incidence has been reported 21 per 100000 populations, however, in different parts of Iran, the incidence varied from “1.5 to 107.5” per 100000 populations (15–17). Although few reports have been published on brucellosis prevalence in some parts of Iran, there is no precise data regarding this disease in country. In addition, aware of disease prevalence in a region will help health decision makers to efficiently prevent and eradicate brucellosis at due time. In this context, we decided to compare the frequency of brucellosis cases in some Iranian provinces to find out the provinces with higher risk of brucellosis incidence. Thus, in the current descriptive study, data was collected from 17103 brucellosis suspected patients referred to Noor Pathobiology Laboratory from 18 out of 31 provinces of Iran during 2013–2015. The samples were analyzed by three different methods (STA, ELISA, and PCR). 15.4% of included patients had brucellosis.

The prevalence rate of the disease in Iran was 0.5% to 10.9% in different provinces (18). Compatible with our finding Hamadan Province had the highest incidence with 107.5 per 100000 populations (16). In addition, brucellosis was mainly found in adult male patients aged 20–39 yr old (41%) which of them 67% were male. In a published report, brucellosis patients had 31.3 yr median age (16,19–21). About 2% of studied samples were positive for *Brucella* DNA using PCR method. Due to higher sensitivity of molecular test compared to serologic assays, it could be explained by sampling time. Regarding molecular detection of bacteria DNA, febrile phase of disease is important for detection of DNA of bacteria in the serum which will be negative after passing viremia time. The lower percentage of positive cases by molecular PCR test can be originated by inappropriate sampling time.

Seasonal incidence of brucellosis in Iran during spring, summer, autumn, and winter were estimated as of 34.4%, 33.2%, 16.4% and 14.9%, respectively (22) that totally are compatible with our findings. Brucellosis prevalence in different provinces of Iran had an ascending trend from 2001 to 2005 (20). However, the prevalence of brucellosis decreased from 17.1% in 2006 to 8.2% in 2009 (16).

## Conclusion

Brucellosis is still considered as an important infectious disease with a high prevalence in many provinces of Iran. It is necessary to implement a national brucellosis control program by increasing medical education, public knowledge and various controlling plans for preventing, controlling and eradicating of brucellosis.

## Ethical considerations

Ethical issues (Including plagiarism, informed consent, misconduct, data fabrication and/or falsification, double publication and/or submission, redundancy, etc.) have been completely observed by the authors.
